# Segmental Duplication of Chromosome 11 and its Implications for Cell Division and Genome-wide Expression in Rice

**DOI:** 10.1038/s41598-017-02796-9

**Published:** 2017-06-02

**Authors:** Rong Zhang, Chao Xue, Guanqing Liu, Xiaoyu Liu, Mingliang Zhang, Xiao Wang, Tao Zhang, Zhiyun Gong

**Affiliations:** Jiangsu Key Laboratory of Crop Genetics and Physiology/Co-Innovation Center for Modern Production Technology of Grain Crops, Key Laboratory of Plant Functional Genomics of the Ministry of Education, Yangzhou University, Yangzhou, 225009 China

## Abstract

Segmental duplication is a major structural variation that occurs in chromosomes. Duplication leads to the production of gene copies with increased numbers of related repeat segments, causing the global genome to be in a state of imbalance. In addition, if the added segment contains a centromeric specific DNA, the duplicated chromosome will have structural multiple centromeres. We identified a segmental duplication containing structurally tricentric regions derived from the short arm of chromosome 11 (11L∙ + 11L∙ + 11S∙11S∙11S∙11S, “∙” represents the centromeric DNA repeat loci), and analyzed its implications for cell division and genome-wide expression. In the variant, only the middle centromere of 11S∙11S∙11S∙11S is functionally active. As a result, the structurally tricentric chromosome was stable in mitosis, because it is actually a functional monocentric chromosome. However, the structurally tricentric chromosome, which usually formed a bivalent, was either arranged on the equatorial plane or was lagging, which affected its separation during meiosis. Furthermore, RNA-seq and RT-qPCR analysis showed that the segmental duplication affected genome-wide expression patterns. 34.60% of genes in repeat region showed positive dosage effect. Thus, the genes on chromosome arm 11S-2 didn’t exhibit obviously dosage compensation, as illustrated by no peak around a ratio of 1.00. However, the gene dosage effect will reduce after sexual reproduction of a generation.

## Introduction

In higher eukaryotes, gene rearrangement, which occurs due to DNA damage repair, exchange and transposon translocation, results in variations in chromosome structure. The main types of variation include deletion, duplication, translocation and inversion of chromosomes^1–3^. Duplication, the addition of the same segment on a chromosome, is a common type of structural variation. This process leads to the presence of duplicate genes in related repeat segments^4–6^. Gene duplication is an important source of genome evolution in eukaryotes^7–9^.

Segmental duplication of chromosomes can disrupt the genome balance. The gene balance hypothesis is often mentioned in reports examining global gene expression in aneuploids^10^, because aneuploidy results in genome imbalance^11^. Gene expression in aneuploids in a variety of species can lead to many different types of responses^12^. Analysis of individual gene suggests that there are two types of effects of aneuploidy: the gene dosage effect and the compensation effect which have been found in budding yeast, maize and *Drosophila* aneuploids^11, 13, 14^. The gene dosage effect indicates that the doses of many genes have been altered in aneuploidy cells, which usually has a negative effect on organism growth and development. Dosage compensation is a regulatory process that ensures that aneuploids have equal amounts of the added gene products^12, 15, 16^. In theory, segmental duplication of a chromosome should lead to both the gene dosage effect and the compensation effect because segmental duplication results in genome imbalance, as in aneuploids. However, there are few reports examining global gene expression patterns following segmental duplication of chromosomes.

On the other hand, genome rearrangements might cause duplication of chromosome(s), which contain multiple centromeric specific DNA^17–19^. The centromere is an essential element of normal chromosomes in eukaryotes. During cell division, each chromosome has only a single functional centromeric region in order to ensure accurate division of the chromosome^20–22^. The plant centromere is a complex composed of DNA sequences and proteins^23–27^. Whereas DNA sequences include large diversity among different species, centromere-specific proteins are relatively conserved^28, 29^. The presence of centromere-specific histone H3 (CENH3) in an active centromere ensures that a chromosome will be correctly transmitted during cell division in plants^30^. In previous studies, multicentric chromosomes were found to be cytologically unstable and could undergo breakage–fusion–bridge cycles in maize, budding yeast and *Drosophila* ^18, 19, 31, 32^. Multicentric chromosomes have been found to be stable in some model plant species, recently^33–37^, because the structurally multicentric chromosomes behave and separate as a functional monocentric chromosome in cell division^31^. What is more, inactive centromeres adopt a heterochromatic structure in plants^31^. Few studies have examined the function of centromere during cell division in duplicate chromosomes containing multiple centromeric specific DNA. How the chromosomes, which contain more than two copies of duplicate segments, pairing is still unknown.

Rice (*Oryza sativa* L.) is a model plant that has been extensively used in molecular biological studies in monocots^38^. The rice genome has been fully sequenced, enhancing global genome expression studies in rice^39, 40^. In addition, well-spread pachytene or prometaphase rice chromosomes are relatively easy to prepare, making rice a good system for studying structural variations and centromere structure in chromosomes as well^41, 42^. Furthermore, rice centromeres DNA are occupied by CentO (a 155-bp satellite repeat) and CRR (a centromere-specific retrotransposon). Between these two DNA elements, the CentO satellite may be key for rice centromere function. It is quantitatively variable among the 12 different chromosome in rice^24^. Although the copy numbers of CentO are different, the amounts of CENH3 that bind functional centromeres are similar in all 12 centromerers^43^. Among the 12 chromosomes centromeric regions, the 5S rDNA, which occupies a single locus very close to the centromere of short arm of chromosome 11, served as a good marker for the identification of chromosome 11^44^. Although CentO and 5S rDNA are overlapped on the centromeres of chromosome 11 at prometaphase^37^, the 5S rDNA sequence was not associated with CENH3 by ChIP-seq^45^.

In our present study, we obtained a new rice chromosome variant exhibiting segmental duplication of short arm of chromosome 11. We analyzed the stability and cytological behavior of the chromosome harboring multiple centromeres and duplicated segments. In our previous research, we found that more copies of genes result in more transcripts, which may ultimately impact on plant genome^46^. We therefore analyzed the global gene expression patterns of the variant by RNA-seq. Understanding the characteristics of segmental duplication would provide a theoretical basis for further analysis of the reasons for the existence of repeat sequences and pseudogenes in eukaryotic genomes, and it would help to confirm that organisms have self-repair and self-regulated ability for the segmental duplications.

## Materials and Methods

### Plant materials

Rice lines Zhongxian 3037 (normal diploid), T1035 (selected from the progeny of a rice line telotrisomic for the short arm and with the long arm of Chr11 broken), YZG-5 (containing a duplicated segment on the short arm of Chr11) and the progeny from YZG-5.

### FISH analysis

Chromosome preparation and FISH analysis of the chromosomes were performed as described in Gong *et al*.^47^. Slides containing chromosomes were incubated with digoxigenin-11-dUTP- and biotin-16-dUTP-labeled probes. The probes were detected using anti-digoxigenin-rhodamine (Roche Diagnostics) and Alexa Fluor 488 streptavidin (Invitrogen). The chromosomes were counterstained with 4′, 6-diamidino-phenylindole (DAPI) in anti-fade solution (Vector Laboratories). Chromosome images were captured under an Olympus BX60 fluorescence microscope using Olympus cellSens Dimension software.

### Anti-OsCENH3 antibody preparation and immunofluorescence assay

The polyclonal anti-OsCENH3 antibody was prepared with a similar approach as described in previous reports^43^. A peptide representing the 14 most N-terminal amino acids conjugated with a cysteine (C-AEPKKKLQFERSPR) was synthesized to be injected into two rabbits. After 7 times of immunoreaction, the whole blood of the rabbits was purified into antisera (AbmartInc). We used the resulting antisera to perform ChIP and FISH assay to confirm the effectiveness of the polyclonal antibody. The results showed the anti-OsCENH3 antibody worked successfully (Figure S1).

The immunofluorescence assay of mitotic chromosomes was performed as described in Gong *et al*.^17^. Slides were incubated in a humidified chamber at 37 °C for 4 h in above anti-OsCENH3 antibody diluted 1:500 in TNB buffer (0.1 M Tris-HCl, pH 7.5, 0.15 M NaCl and 0.5% blocking reagent). After three rounds of washing in PBS buffer, the slides were incubated with Goat anti-rabbit Alexa Fluor 488 antibody (1:500; Invitrogen). The chromosomes were counterstained with DAPI in anti-fade solution (Vector Laboratories). Chromosome images were captured under an Olympus BX60 fluorescence microscope using a cooled CCD camera (Olympus, DP80).

### Real-time quantitative PCR (qPCR) and reverse transcription quantitative PCR (RT-qPCR)

Real-time quantitative PCR analysis was performed using the ABI ViiA^TM^ Real Time Quantitative PCR System with SYBR Premix Ex Taq (TaKaRa) and gene-specific primers (Table S1). LOC_Os08g21690 was selected as an internal reference gene. DNA was extracted from the leaves of normal and variant plants. Data analysis was performed after the completion of qPCR. The relative amounts of specific chromosome regions were quantified using the 2^−ΔΔCT^ method, where ΔΔCT is the difference between the threshold cycles of the test and the starting copy number of the DNA fragment. ΔΔCT = ΔCT (target DNA) − ΔCT (DNA from the original line): ΔCT (target DNA) is the difference in threshold cycles between the target DNA region and the reference DNA region, ΔCT (DNA from the original line) is the threshold cycles of the original DNA region subtracted from the threshold cycles of the reference DNA region^48^. The mean threshold cycle values were calculated from four experiments, and each DNA sample was subjected to qPCR analysis in triplicate. The 2^−ΔΔCT^ values of each DNA fragment were compared.

RT-qPCR was performed with the ABI ViiaTM Real Time Quantitative PCR System and SYBR Premix Ex Taq (TaKaRa) using gene-specific primers (Table S2) and the LOC_Os01g50622 gene as an internal reference for normalization. Total RNA was extracted from leaves of 14-day seedlings and roots with RNAsimple Total RNA Kit (TIANGEN) and cDNA was synthesized from the RNA with FastQuant RT Kit (With gDNase) (TIANGEN) according to the manufacturer’s instructions.

### Tissue isolation and RNA extraction for RNA-seq

All tissue samples were collected from 14-day-old rice seedlings grown in culture tubes. The plants were grown in a growth chamber under a cycle of 12 h light at 25 °C followed by 12 h dark at 25 °C. All leaves of the seedlings were collected under a clean bench and frozen immediately in liquid nitrogen. Total RNA was extracted and sequenced by Shanghai Biotechnology Corporation (contract number: BC14374-1). The leaf transcriptome was analyzed using Illumina HiSeq 2500. We generated 97.08 million and 93.38 million RNA-seq reads from Zhongxian 3037 (control) and the variant (YZG-5), respectively. We mapped 76.63% of the Zhongxian 3037 RNA-seq reads and 78.09% of the variant reads to the TIGR7 reference genome using TopHat2^49^. We used Cufflink^50^ to measure the expression level (FPKM) of rice annotated genes (TIGR7, http://rice.plantbiology.msu.edu/).

## Results

### Origin and molecular cytological examination of YZG-5

**Figure 1:**
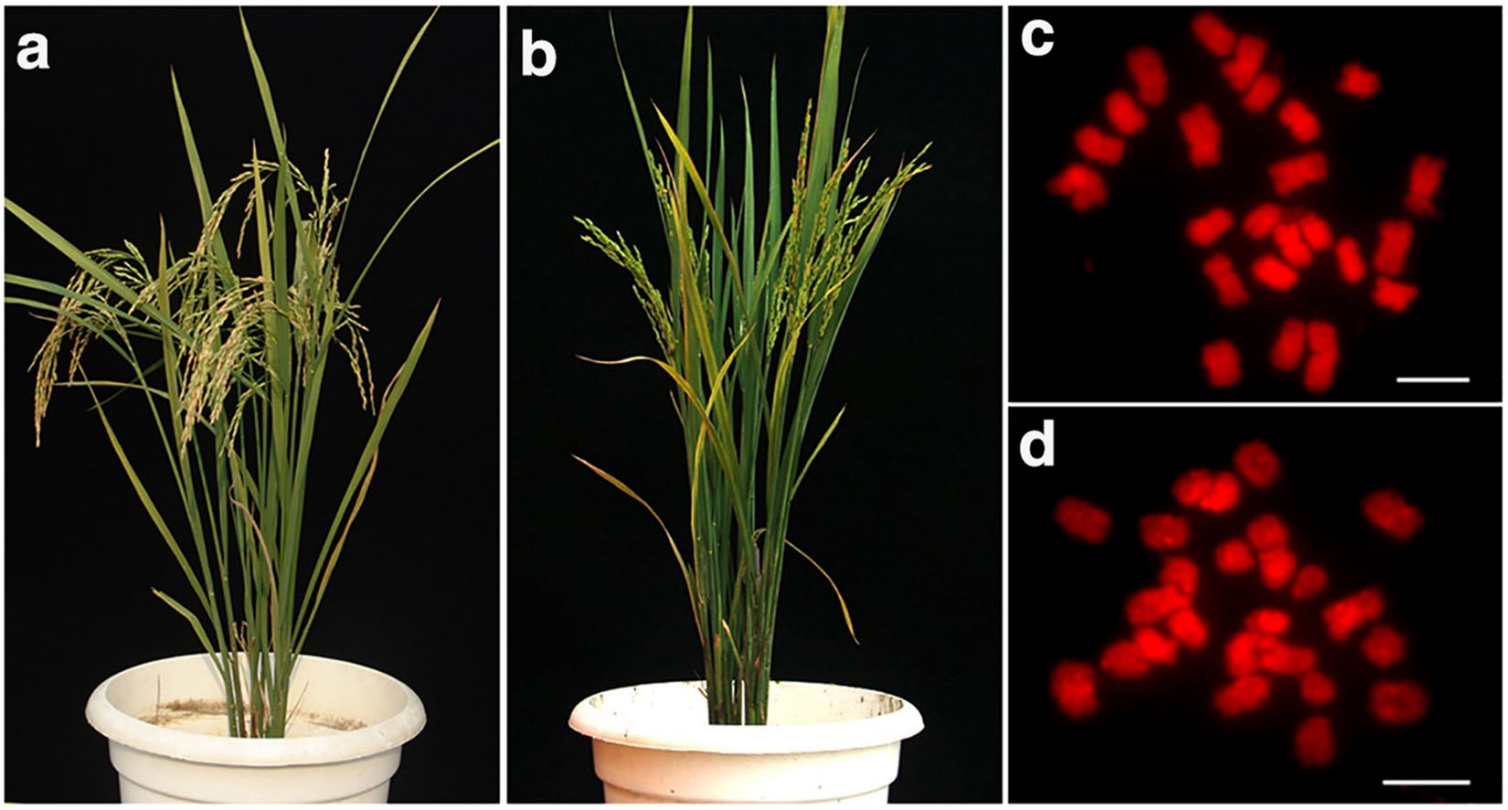
Morphological traits of rice line Zhongxian 3037 and variant YZG-5. (**a**) Zhongxian 3037. (**b**) Variant YZG-5 exhibits deep green leaf color and poor seed setting. (**c**) Zhongxian 3037 has 24 chromosomes in each somatic cell. (**d**) YZG-5 has 25 chromosomes in each somatic cell.

**Figure 2:**
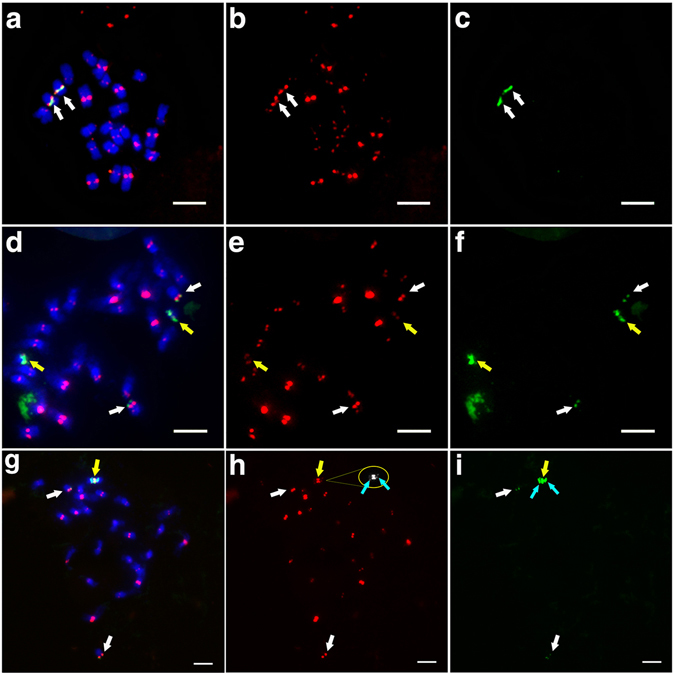
FISH analysis of rice line Zhongxian 3037 and variant YZG-5. Chromosomes were counterstained with DAPI. Red indicates CentO signals, green indicates 5S rDNA signals. Scale bars represent 5 μm in all images. (**a**–**c**) Normal chromosome 11 in line 3037, with two pairs of CentO and 5S rDNA signals. (**d**–**f**) Line T0135, with two pairs of telocentric chromosome 11. White arrows show 11L∙ with weaker 5S rDNA signals and stronger CentO signals, and yellow arrows show 11S· with stronger 5S rDNA signals and weaker CentO signals. (**g**–**i**) Variant YZG-5, with a structurally tricentric chromosome and two telocentric chromosomes derived from Chr11. White arrows show 11L∙ with weaker 5S rDNA signals and stronger CentO signals. In figure **h**, yellow and cyan arrow indicate 11S∙11S∙11S∙11S with one stronger CentO signal located at the middle region and two weaker CentO signals, respectively. In figure **i**, yellow and cyan arrows indicate 11S∙11S∙11S∙11S with three stronger 5S rDNA signals.

YZG-5 is a morphological variant discovered from the inbred progenies of a rice line telotrisomic for chromosome 11 (Chr11), which was derived from *indica* rice variety Zhongxian 3037. Compared to Zhongxian 3037 (Fig. 1a), the variant plant had deep green leaves and a poor rate of seed set of only 10.5% (Fig. 1b). Based on cytological analysis of the variant at mitosis, we found that the chromosome number of the variant was 25 in all somatic cells examined (Fig. 1d), whereas that of the normal diploid was 24 (Fig. 1c). As YZG-5 was derived from the progeny of Chr11 variants, we used 5S rDNA, as a probe for FISH analysis. The 5S rDNA signals overlapped with those of a rice centromere-specific DNA sequence (CentO) in the normal diploid (white arrows in Fig. 2a–c), indicating that the 5S rDNA was located at the centromere region of Chr11 close to its short arm. To further differentiate between the short arm and the long arm of Chr11 according to the 5S rDNA and CentO signals, we selected another variant, T0135, in which Chr11 had been broken, forming two types of telocentric chromosomes. One type is the long arm of Chr11 (designated 11L∙; “∙” represents the centromeric repeat loci), with stronger CentO signals (white arrows in Fig. 2d,e) and weaker 5S rDNA signals (white arrows in Fig. 2d,f). The other type is the short arm of Chr11 (designated 11S∙; “∙” represents the centromeric repeat loci), with weaker CentO signals (yellow arrows in Fig. 2d,e) and stronger 5S rDNA signals (yellow arrows in Fig. 2d,f). Therefore, we used 5S rDNA and CentO as cytological markers, not only to identify the centromere region of Chr11, but also to distinguish 11S∙ from 11L∙.

The labeled probes 5S rDNA and CentO were hybridized (by FISH) to prometaphase chromosomes of YZG-5. The results show that there were three chromosomes with 5S rDNA signals in the somatic cells of YZG-5. Two chromosomes with weaker 5S rDNA signals and stronger CentO signals located at the telomere region were 11L∙ (white arrows in Fig. 2g–i); the third chromosome had three stronger 5S rDNA signals (yellow and cyan arrows in Fig. 2i) and three CentO signals (yellow arrows in Fig. 2g), in which two CentO signals were very weak (showed in circle and cyan arrows in Fig. 2h) and the middle signal was stronger (yellow arrow in Fig. 2h). Total signals did not localize to the telomere region of this chromosome. These results indicate that this chromosome contains more than one short arm of Chr11, as shown in the model image in Fig. 2g. This preliminary analysis suggests that YZG-5 contains the segmental duplication derived from short arms of Chr11 and have three centromeric DNA repeat loci, which is a structurally tricentric chromosome.

### Source of segmental duplication of the variant YZG-5

**Figure 3:**
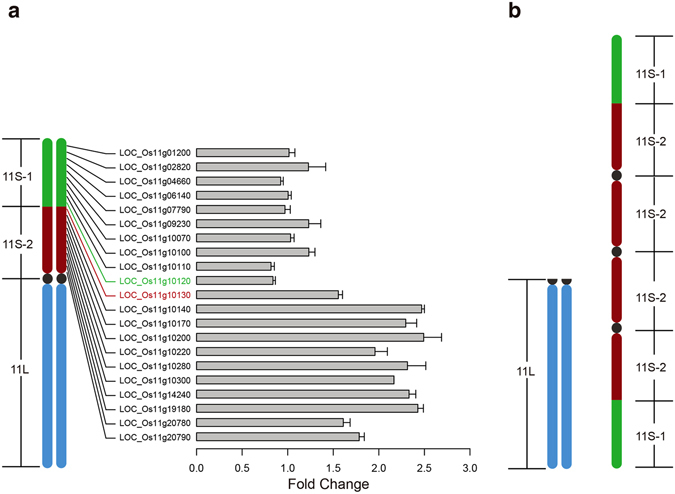
Real-time quantitative PCR analysis and model of Chr11 in YZG-5. (**a**) Diagram of Chr11 with 21 related genes uniformly distributed on the short arm and qPCR results. The level of gene expression differed almost two-fold between Os11g01200-11g10130 and Os11g10120-11g20790. (**b**) Model pattern of the composition of Chr11 in variant YZG-5.

To further clarify the source of the duplicate segments in YZG-5, we conducted qPCR analysis of this variant. If this variant contains two extra segments derived from the short arms of Chr11, the number of specific DNA sequences located on the extra segment would be double that of the normal diploid. To investigate this possibility, we designed 21 specific primer pairs for 21 genes that are uniformly distributed on the short arm of Chr11 (Fig. 3a).

Using these primers for qPCR, we found that the amount of DNA amplified from gene LOC_Os11g01200 (close to the telomeric region of the short arm of Chr11) to LOC_Os11g10120 (close to the middle region of the short arm of Chr11) was not significantly different between the normal diploid and the variant YZG-5 (Fig. 3b), with a ratio near 2:2. However, the amount of DNA amplified from gene LOC_Os11g10130 (close to the middle region of the short arm of Chr11) to LOC_Os11g20790 (close to the centromeric region of the short arm of Chr11) was significantly different between the normal diploid and the variant YZG-5 (Fig. 3a), with a ratio near 2:4. Therefore, the repeat region of YZG-5 indeed consists of the two repeat segments from the short arm of Chr11. The breakpoint is between LOC_Os11g10130 and LOC_Os11g10120 (Fig. 3a). According to the TIGR7 database (http://rice.plantbiology.msu.edu/), the repeat segment contains 513 genes from LOC_Os11g10130 to LOC_Os11g20790, accounting for 41.30% of all genes on the short arm of Chr11.

To clarify the composition of Chr11 in variant YZG-5, we used 22 + 11L∙ + 11L∙ + 11S∙11S∙11S∙11S to represent the configuration of the variant’s Chr11 (Fig. 3b), where “22” represents the 22 other chromosomes in the rice genome excluding Chr11 and “∙” represents the centromere. To further distinguish between the non-repeat and repeat segments of the short arms of Chr11, we used 11S-1 and 11S-2 to represent the non-repeat and repeat segment, respectively (Fig. 3b).

### Activity and mitotic behavior analysis of tricentromere in the repeat segment

According to the above analysis, YZG-5 not only contains two repeat segments, but it has three centromeric DNA repeat loci, which is a structurally tricentric chromosome variant. In rice, CENH3 is a key element of a functional centromere that can be used as an identification marker for functional centromeric chromatin^28, 37, 51, 52^. To determine whether the three centromeric DNA repeat loci of the structurally tricentric chromosome in YZG-5 have normal centromeric function, we conducted CENH3 immunofluorescence analysis of somatic cells of YZG-5.

**Figure 4:**
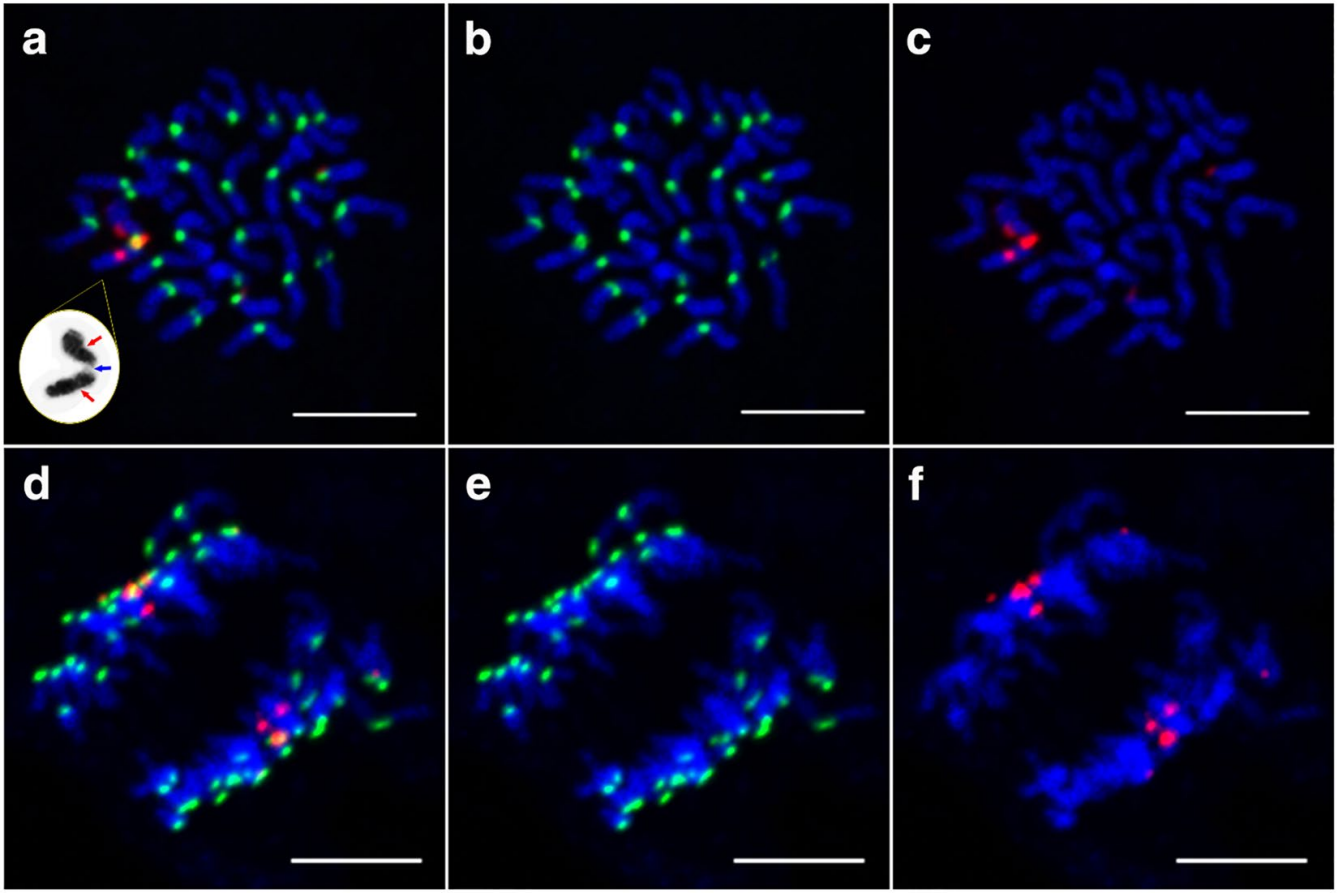
Localization of CENH3 and 5S rDNA during mitosis in variant YZG-5. Chromosomes were counterstained with DAPI. Red signals indicate 5S rDNA and green signals indicate CENH3. Scale bars represent 5 μm in all images. (**a**-**c**) Distribution of CENH3 and 5S rDNA signals on somatic chromosomes at mitosis prometaphase in variant YZG-5. As shown with a yellow arrow, only one CENH3 signal overlaps with the middle 5S rDNA signal on the structurally tricentric chromosome. White arrows indicate 11L∙. In figure **a**, the panel for DAPI stained 11S∙11S∙11S∙11S is indicated by grayscale, with three centromeric regions shown by blue and red arrows. (**d**–**f**) Distribution of CENH3 and 5S rDNA signals on somatic chromosomes at mitosis anaphase. The structurally tricentric chromosome with one CENH3 signal divided normally.

According to CENH3 immunofluorescence analysis, normal chromosomes produced a pair of green signals in the centromere region. If the three centromeric DNA repeat loci were functional, three pairs of CENH3 signals would be detected in a structurally tricentric chromosome. After observing 30 somatic cells at mitosis prometaphase, combined with the 5S rDNA signals, we found that there was only one pair of CENH3 signals overlapped with the middle 5S rDNA signal at the tricentric chromosome (Fig. 4a–c). As above mentioned, three 5S rDNA signals overlapped the three CentO signals at prometaphase in mitosis, respectively (Fig. 2g). The middle 5S rDNA signal location was the middle centromeric region, which contained more centromeric DNA sequences. Therefore, we judged one centromere contains CENH3, which is located at the middle position of the structurally tricentric chromosome (11S∙11S∙11S∙11S). In addition, this middle centromere shows primary constriction (blue arrow in Fig. 4a), whereas the other two contain centromeric DNA but no CENH3 and obvious constriction (red arrows in Fig. 4a). The same results were observed at anaphase in mitosis (Fig. 4d–f). To analyze the structurally tricentric chromosome behavior at different stages of mitosis, we observed 62 cells at each stage of mitosis, finding that all structurally tricentric chromosomes divided normally, as did the other normal chromosomes (Figure S2). Therefore, the structurally tricentric chromosome contains only one functional centromere region, which maintains the stability of chromosome separation during mitosis, and is a functional monocentric chromosome.

### Meiotic behavior of repeat segments in YZG-5

**Figure 5:**
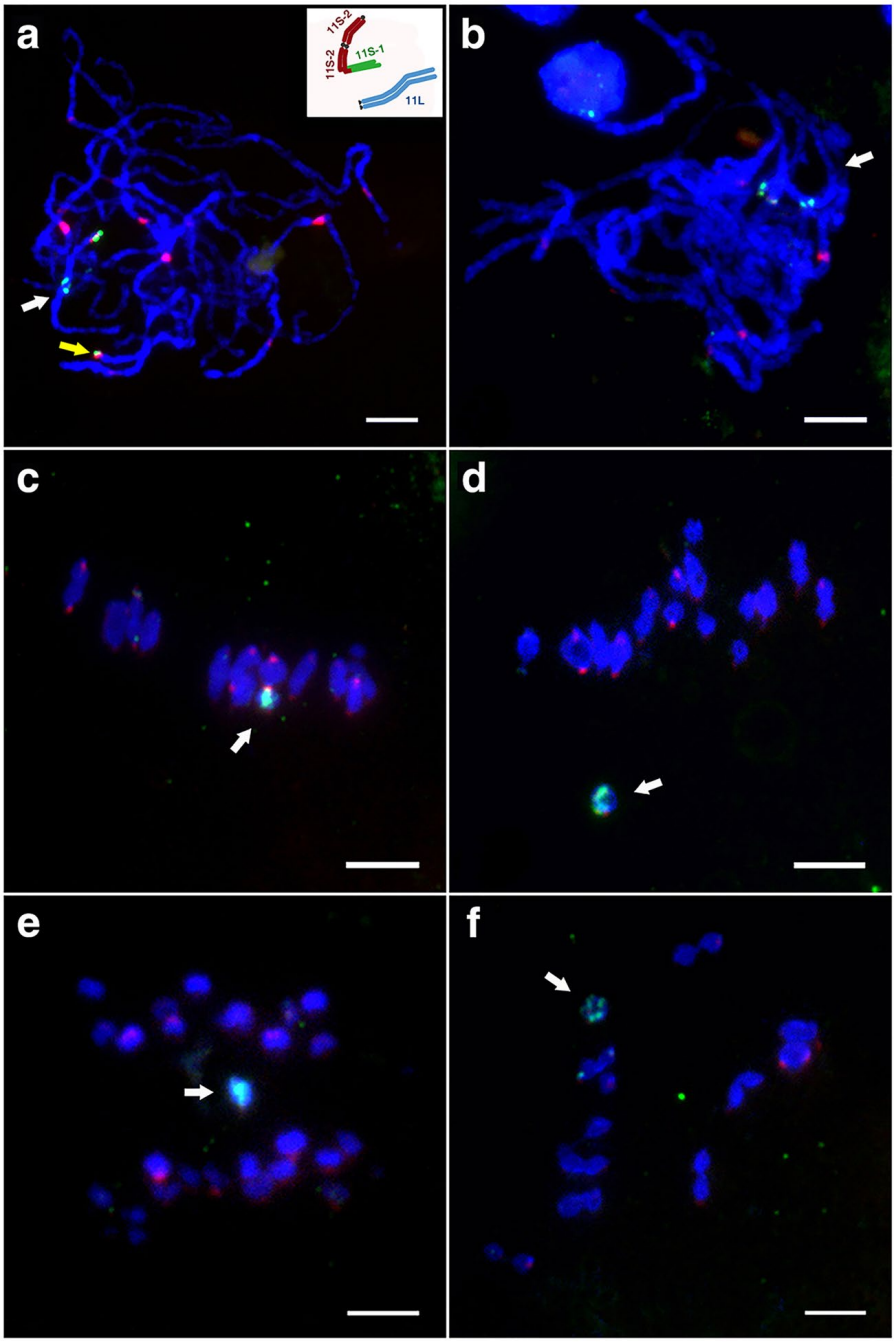
Behavior of the structurally tricentric chromosome at meiosis in variant YZG-5. Chromosomes were counterstained with DAPI. White arrows show the structurally tricentric chromosome 11S∙11S∙11S∙11S. Red signals indicate CentO and green signals indicate 5S rDNA. Scale bars represent 5 μm in all images. (**a**,**b**) Synapsis of the 11th homologous chromosomes at meiotic prophase I. (**a**) The structurally tricentric chromosome 11S∙11S∙11S∙11S (white arrow) and two 11L∙ (yellow arrow) exhibited bivalents respectively, with the illustration of the state of 11S∙ and 11L∙. (**b**) Synapsis of the chromosome 11S∙11S∙11S∙11S indicated no obvious bivalent structure. (**c**) The structurally tricentric chromosome with 5S rDNA signal did not divide at meiotic metaphase I. (**d**) The structurally tricentric chromosome with 5S rDNA signal was not involved in the arrangement of chromosomes on the equatorial plate at meiotic metaphase I. (**e**) At meiotic anaphase I, the lagging chromosome was on the equatorial plate. (**f**) At meiotic anaphase I, the structurally tricentric chromosome was distributed to one daughter cell.

Meiosis, other cell division, is an important process of gamete formation. During normal meiosis, two homologous chromosomes must undergo pairing and synapsis^53, 54^. As described above, YZG-5 contains four of the same repeat segments from 11S∙ (Fig. 3d). To analyze the pairing behavior of this abnormal chromosome (11S∙11S∙11S∙11S), which contains three centromeres, we conducted FISH analysis using CentO and 5S rDNA in pollen mother cells of YZG-5. In 23 of the 25 cells observed, the structurally tricentric chromosome paired with itself, forming a bivalent between the four repeated segments from the short arms at pachytene stage (Fig. 5a). In all of these cells, the functional centromere was located at one end of the bivalent. In the two remaining cells, synapsis of the abnormal chromosome was irregular, and there was no obvious bivalent structure (Fig. 5b).

We also investigated the behavior of the abnormal chromosome, 11S∙11S∙11S∙11S, at other stages of meiosis. We observed two types of behavior by the abnormal chromosome at metaphase I. Sometimes the abnormal chromosome was arranged at the equatorial zone together with other chromosomes (Fig. 5c). At other times, we observed a lagging phenomenon, as the abnormal chromosome was not involved in the arrangement of chromosomes on the equatorial plate (Fig. 5d). Of the 24 cells observed at meiotic metaphase I, 14 cells exhibited the former behavior and 10 exhibited the latter. During meiotic anaphase I, the lagging chromosome was present alone on the equatorial plate and would probably have been lost (Fig. 5e). In another instance, the abnormal chromosome was distributed to one daughter cell, as 11S∙11S∙11S∙11S + 11L∙ (Fig. 5f). That is, the presence of this tricentric chromosome led to the production of abnormal gametes, which might have influenced the chromosome characteristics of the progeny of YZG-5.

### Genome-wide analysis of expression characteristics in YZG-5

**Figure 6:**
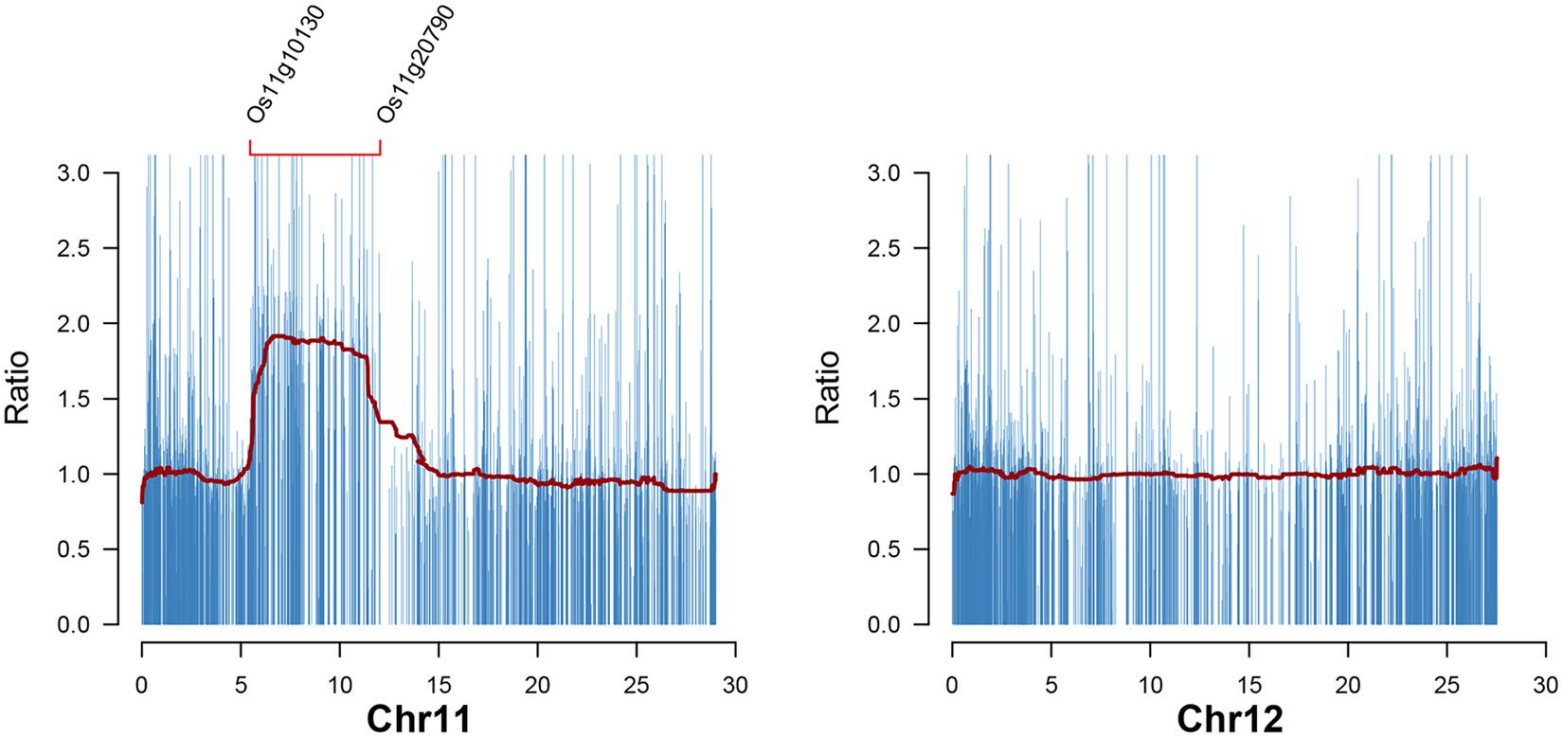
Gene expression level between Chr11 and Chr12 in YZG-5. Blue bar represents expression level change of each expressed gene (FPKM > 0) in Chr11 and Chr12 of YZG-5. Red lines represent median score of expression level change in 100 genes sliding windows. X-axis represent genes loci. The region from Os11g10130 to Os11g20790 represents 11S-2.

**Figure 7:**
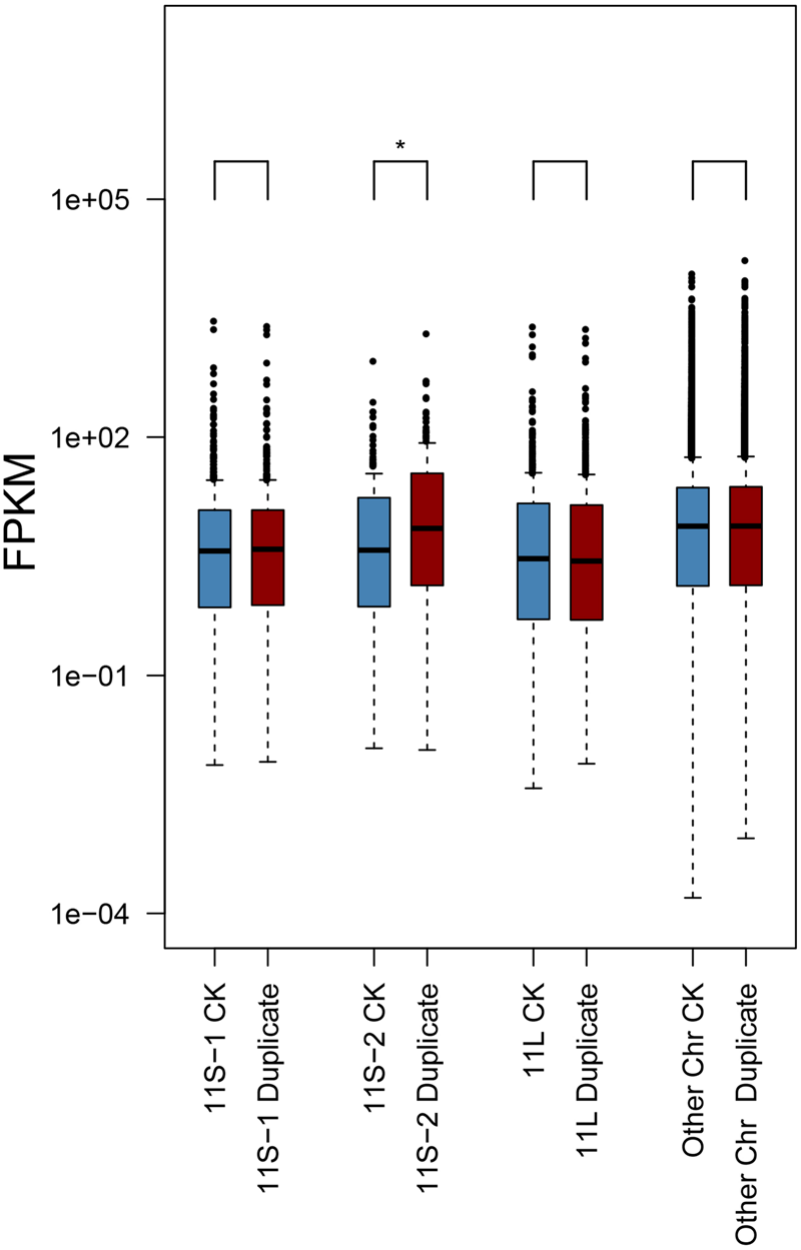
*Kolmogorov-Smirnov* tests of genes expression level between the normal diploid and 11S-2 region. Y-axis represent genes expression level change.

In theory, segmental duplication of a chromosome may lead to gene dosage effect and compensation effect^10–12^. In order to investigate genes dosage/compensation effect in both duplicated regions and other normal regions, we compared the genes expression level between YZG-5 and the normal diploid by applying high-throughput RNA-seq experiments. First, a ratio score (YZG-5/the normal diploid) plot of all expressed genes in whole rice genome. In Fig. 6, blue bars represent expressed genes expression level change between YZG-5 and the normal diploid. Red lines correspond to median ratio score of 100 genes sliding window. Our result showed that genes expression level from 5.4 M to 12.1 M of Chr11 in YZG-5 is twice as the normal diploid. On the other hand, the ratio scores of other chromosome and rest region of Chr11 is around 1.0 (Fig. 6 and Figure S3). Furthermore, we found that the genes expression level is significant higher in YZG-5 compared with normal diploid in 11S-2 region (p < 0.004, *Kolmogorov-Smirnov* tests). In addition, we observed that genes expression level in rest of Chr11 (11S-1, p < 0.726; 11L, p < 0.673, *Kolmogorov-Smirnov* tests) and other chromosomes (p < 0.514, *Kolmogorov-Smirnov* tests) are insignificant different (Fig. 7). Thus, the expression of other genes, located on the no-repeat regions, did not obviously differ at the genome level between the variant and the normal diploid, although the variant had both up-regulated (YZG-5/the normal diploid ≥ 2) and down-regulated (YZG-5/the normal diploid ≤ 0.5) genes at a ratio of 5.55% and 4.18%, respectively (Table 1), implying that segmental duplication of Chr11 has less effect on global genome expression.

**Table 1:** Statistical analysis of differentially expressed genes on each chromosome.

Chr	Number of Expressed Genes	Up Number	Up Ratio (%)	Down Number	Down Ratio (%)
Chr1	3386	179	5.29	145	4.28
Chr2	2763	118	4.27	103	3.73
Chr3	3016	141	4.68	95	3.15
Chr4	2047	121	5.91	78	3.81
Chr5	1952	91	4.66	70	3.59
Chr6	1927	99	5.14	76	3.94
Chr7	1789	98	5.48	94	5.25
Chr8	1557	80	5.14	84	5.39
Chr9	1332	73	5.48	48	3.60
Chr10	1223	77	6.30	62	5.07
11L	646	60	9.29	39	6.04
11S-1	448	37	8.26	23	5.13
11S-2	211	73	34.60	8	3.79
Chr12	1307	64	4.90	61	4.67
Total	23604	1311	5.55	986	4.18

### Characterization of the distribution of differentially expressed genes in repeat segments

**Figure 8:**
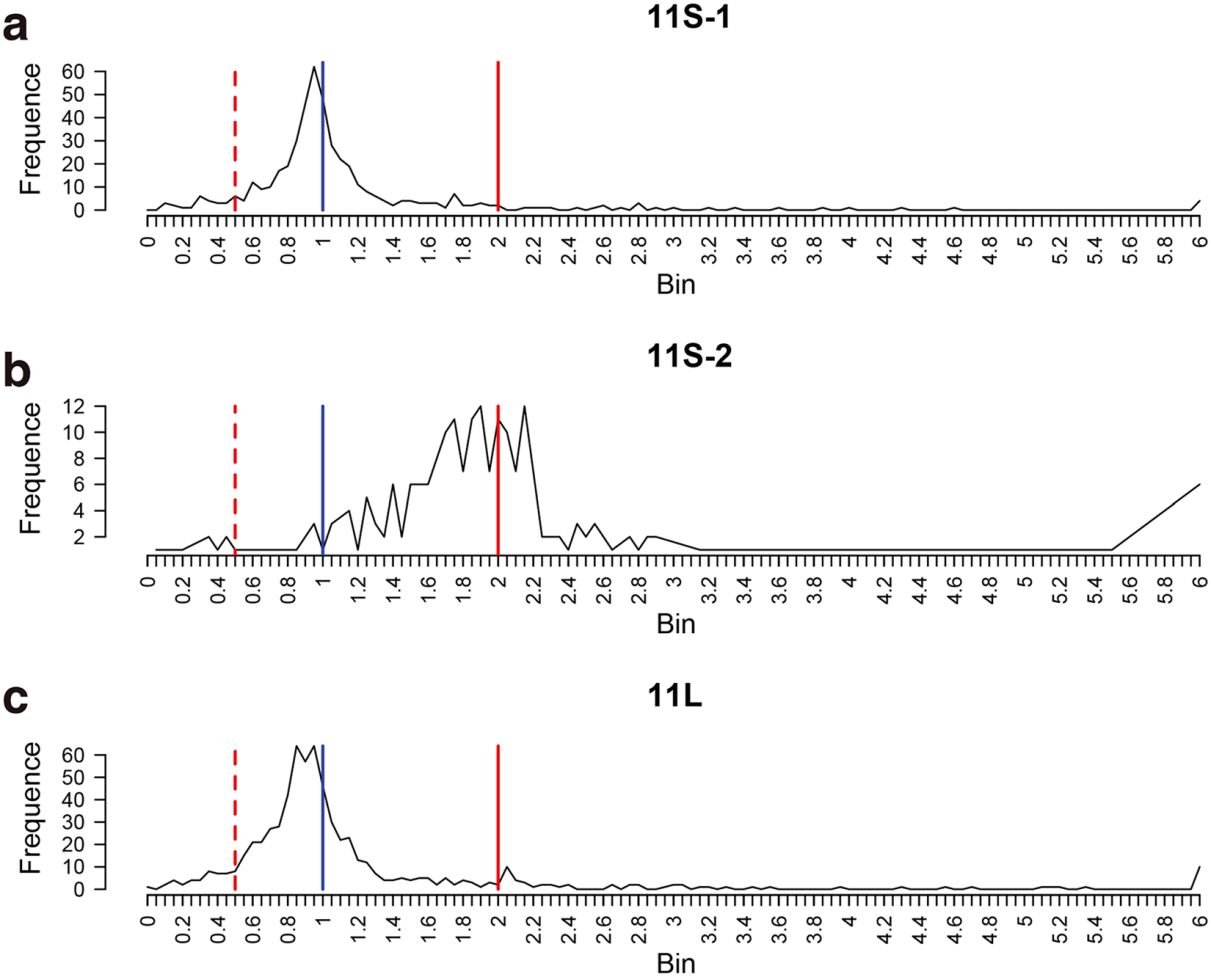
Global gene expression ratio distribution on Chr11 of YZG-5. The blue line represents the ratio 1.0 (no change), and the red solid line represents the ratios 2.0[the ratio of positive dosage effects (4/2)]. The red dashed line represents the ratios 0.5[the ratio of inverse dosage effects (2/4)]. The ratio distributions of gene expression on 11S-1 (**a**) and 11L (**c**) are roughly centered around 1.0, while the gene expression ratio distribution of 11S-2 (**b**) is centered around 2.0.X-axis represents ratio bin, Y-axis represents gene frequency in each bin.

To further clarify the levels of gene expression on each chromosome, we determined how many differentially expressed genes on each chromosome accounted for the total number of expressed genes on the corresponding chromosome (Figure S4 and Table 1). Chromosome 11 of variant YZG-5 can be categorized as 11L∙, 11S-1 (non-repeat section) or 11S-2 (repeat section) based on composition. Then, we performed a ratio distribution analysis for expression level (FPKM) of expressed gene within three regions of Chr11, by following previous research^55^. The ratios were plotted with bins of 0.05 increments. Based on the distribution analysis, 34.60% (37/448) genes’ ratio score is greater than 2.0 in 11S-2 region, exhibiting gene dosage effect. It’s significantly higher than 9.29% 11L (60/646, *p* < 2.2 × 10^−15^ Fisher’s exact test) and 8.26% 11S-1 (37/448, *p* < 4.1 × 10^−15^ Fisher’s exact test), which consider as control region in the same chromosome (Fig. 8). Thus, the genes on chromosome arm 11S-2 didn’t exhibit obviously dosage compensation, as illustrated by no peak around a ratio of 1.00.

To verify RNA-seq result, we selected ten genes from 11S-2 region for RT-qPCR analysis. The result shows that four genes fold change of expression level are greater than 2.0 and only one gene fold change of expression level is less than 1.0 (Figure S5a) in seedlings. Both RNA-seq and RT-qPCR results suggest that dosage effect is present on 11S-2 region of variant YZG-5 seedlings. In order to verify if the dosage effect is still present in the progeny of variant YZG-5, we selected the offspring of YZG-5 from sexual reproduction to conduct RT-qPCR analysis. Because the chromosome composition of offspring from YZG-5 is segregated, the offspring individuals, which contained 11L∙ + 11L∙ + 11S∙11S∙11S∙11S similar as YZG-5 parents, were chosen by FISH analysis. Interestingly, the result shows that only one gene fold change of expression level are greater than 2.0 in leaves from the offspring individuals (Figure S5b); the similar result was shown in roots from the same offspring individual (Figure S5c).

## Discussion

### Functional Centromere May Play a More Important Role in Homologous Pairing of Chromosomes with Repeated Segments

In this study, we found that although the tricentric chromosome in YZG-5 contains three centromeric DNA sequences, only one centromeric DNA region contains CENH3. The tricentric chromosome showed stable inheritance during mitosis, which is consistent with reports in maize^35^. We also found that all CENH3 signals were located in the middle centromeres, which contained more centromeric DNA sequences than the others. No CENH3 signals were detected on the two other centromeres, which contained fewer centromeric DNA sequences. Indeed, tricentric chromosomes exist in wheat, and two of the centromeres, which have weak CENH3 signals, are often inactivated^56^.

During meiosis, chromosome pairing and synapsis between homologs occur during early prophase I^53, 54^. When only one pair of homologous chromosomes is present, the homologous chromosomes form bivalents by pairing and synapsis. However, when more than two homologous chromosomes are present, multivalents form between homologous chromosomes^57^. In the present study, chromosome 11S∙11S∙11S∙11S, which contained four homologous regions of 11S-2 in YZG-5, formed a bivalent structure in more than 90% of cells when four homologous segments paired. In all bivalents, the functional centromere was located at the end of the chromosome when the chromosome folded onto itself. Fewer multivalents formed, although there were four homologous regions in which the 2/2 pairing model took priority over the other pairing model.

Pairing requires centromere activity, as the presence of centromeric repeats is not sufficient for pairing^58^. However, little is known about how centromere activity affects homologous chromosome pairing. In this study, we found that a functional centromere tend to guide the pairing of homologous segments, which may play a more important role than the presence of homologous segments. Although such studies are difficult due to limited research materials and available methods, the functional mechanism of centromere activity mediating the pairing of homologous chromosomes requires further study.

### Segmental duplication showed dosage effect and less dosage compensation effect in rice

The gene compensation effect, which reduces the negative effect of aneuploidy, has been observed in *Arabidopsis*, *Drosophila*, maize and nematodes^59–61^. In aneuploidy maize, the number of gene copies on the extra chromosome differed from that of normal individuals, but the transcription levels of most genes did not change, showing the dosage compensation effect in the embryo and endosperm tissues of 30 days^13^. In *Arabidopsis*, the trisomy 5 disrupts gene expression throughout the genome at a stage of almost ten rosette leaves^60^. These results suggest that the extra segments can lead to differences in gene copy number at the genome level, but a mechanism might exist to allow mutant individuals to survive by self-regulating gene expression. In this study, we found that 34.60% genes on the 11S-2 region showed gene dosage effect in leaves. Furthermore, the dosage compensation effect was not observed in the same region. Regardless previous reports showed obvious dosage compensation effect on the added chromosomes with the highest peak at a ratio of 1.0^62^, which differ from our results. Through long-term adjustment and evolution, organisms might adapt to the existence of segmental duplication, which shows dosage compensation effect in different species^13, 55^. In the present study, rice seedling from asexual reproduction by tissue culture might be the rapidly evolving stage, so no obvious compensation effect has been shown on the 11S-2 region. To test the hypothesis preliminarily, we conduct the similar RT-qPCR analysis in progeny of YZG-5. Compared with the YZG-5 parents, the number of genes showing gene dosage effect decreased in progeny of YZG-5. Thus, we made a prediction preliminarily that the gene dosage effect will reduce after sexual reproduction of a generation. If there has obvious compensation effect, we will get more generations to research in the future.

The addition of an individual chromosome has much more of an impact on genome-wide expression than the addition of the entire genome in polyploids: the addition of individual chromosome makes the entire genome unbalanced, while polyploid genomes are still in a state of equilibrium^63–65^. Genomic imbalance strongly affects the transcription and expression of the entire genome. The equilibrium of the genome can affect gene expression, quantitative traits and dosage compensation and lead to aneuploidy syndrome^10^. In *Arabidopsis*, segmental duplication of chromosome 5 has an impact on the entire genome in normal diploid and trisomic plants. There were 12–13% of transcripts across all chromosomes that were up-regulated with respect to their chromosomal neighborhoods. Down-regulation on other chromosomes was only observed for 8–9% of transcripts^60^. In the present study, we found that segmental duplication of Chr11 affected genome-wide expression in rice, which revealed both up-regulated and down-regulated genes at a ratio of 5.55% and 4.18%, respectively. The impact probability is very low which explain the segmental duplication of 11S∙11S∙11S∙11S has less impact on the global gene expression.

## Electronic supplementary material

Supplementary information

Table S1

Table S2
