# Supplementary material for: Segmental Duplication of Chromosome 11 and its Implications for Cell Division and Genome-wide Expression in Rice
Source: Sci Rep. 2017 Jun 2;7:2689. doi: 10.1038/s41598-017-02796-9 (PMC5457480; doi:10.1038/s41598-017-02796-9)
Supplement: Supplementary file 1 — Supplementary information [file 41598_2017_2796_MOESM1_ESM.pdf]

**Segmental Duplication of Chromosome 11 and its Implications for Cell Division and Genome-wide Expression in Rice**

Rong Zhang, Chao Xue, Guanqing Liu, Xiaoyu Liu, Mingliang Zhang, Xiao Wang, Tao Zhang*, Zhiyun Gong*

Jiangsu Key Laboratory of Crop Genetics and Physiology/Co-Innovation Center for Modern Production Technology of Grain Crops, Key Laboratory of Plant Functional Genomics of the Ministry of Education, Yangzhou University, Yangzhou 225009, China

*For correspondence (e-mail: zygong@yzu.edu.cn or zhangtao@yzu.edu.cn).

**Supplementary Information**

**Figure S1.** The immunofluorescence analysis of OsCENH3 and FISH analysis of ChIP-DNA immunoprecipitated with anti-OsCENH3 antibody in rice.

**Figure S2.** Chromosome behavior at each stage of mitosis in variant YZG-5.

**Figure S3.** Gene expression level of each chromosome in YZG-5.

**Figure S4.** Ratio of differentially expressed genes to total genes on the corresponding chromosome.

**Figure S5.** The expression (fold change) values of ten genes on 11S-2 region in the seedlingsof YZG-5and the seedlings and roots ofYZG-5 offsprings.

**Supplementary Table S1.** Primer pairs used for qPCR analysis of relative amounts of specific chromosome regions.

**Supplementary TableS2.** Primer pairs used for RT-qPCR analysis of ten genes on 11S-2 region.

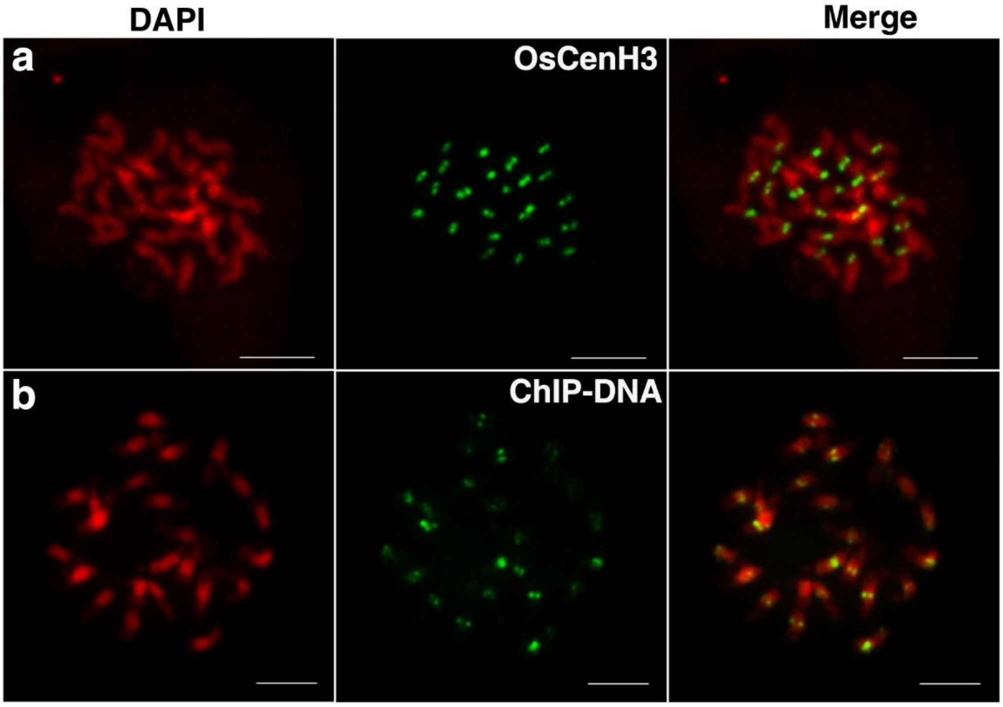


**Figure S1. The immunofluorescence analysis of OsCENH3 and FISH analysis of ChIP-DNA immunoprecipitated with anti-OsCENH3 antibody in rice.**

Chromosomes were counterstained with DAPI, indicated by red signals, and green signals indicate OsCENH3. Scale bars represent 5 μm in all images. (**a**) The immunofluorescence analysis showed the signals of anti-OsCENH3 antibody were almost located at the centromere region of each chromosome. (**b**) FISH results showed the ChIP-DNA immunoprecipitated with anti-OsCENH3 antibody was located at the centromere region of each chromosome in rice.

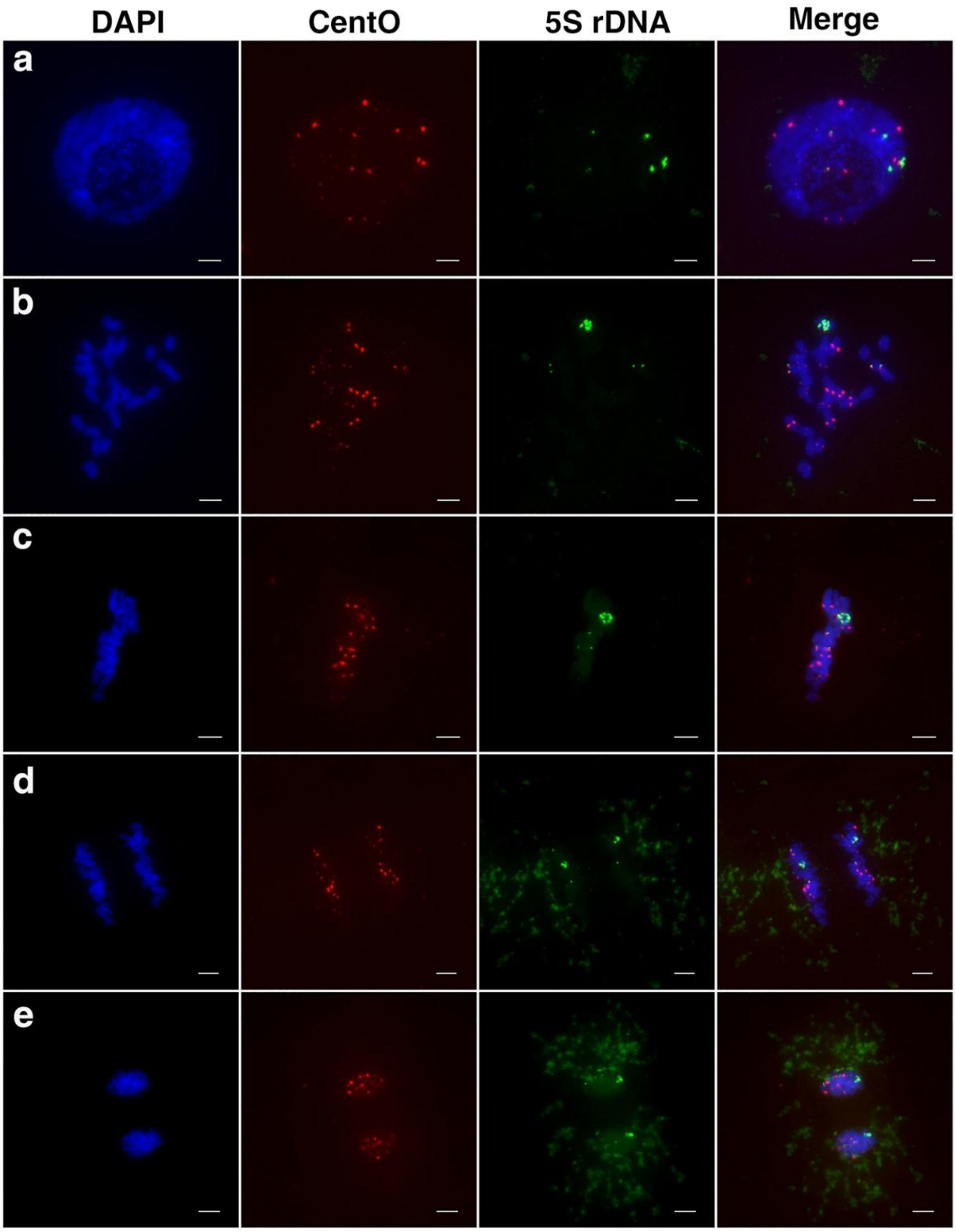


**Figure S2. Chromosome behavior at each stage of mitosis in variant YZG-5**

Chromosomes were counterstained with DAPI. Red signals indicate CentO and green signals indicate 5S rDNA. Scale bars represent 5 μm in all images. (**a**-**b**) Mitotic interphase and prophase. (**c**) Mitotic metaphase. (**d**-**e**) Mitotic anaphase and telophase. At each stage of mitosis, the 5S rDNA signals divided normally, indicating that the tricentric chromosome 11S•11S•11S•11S has only one functional centromere at the middle position.

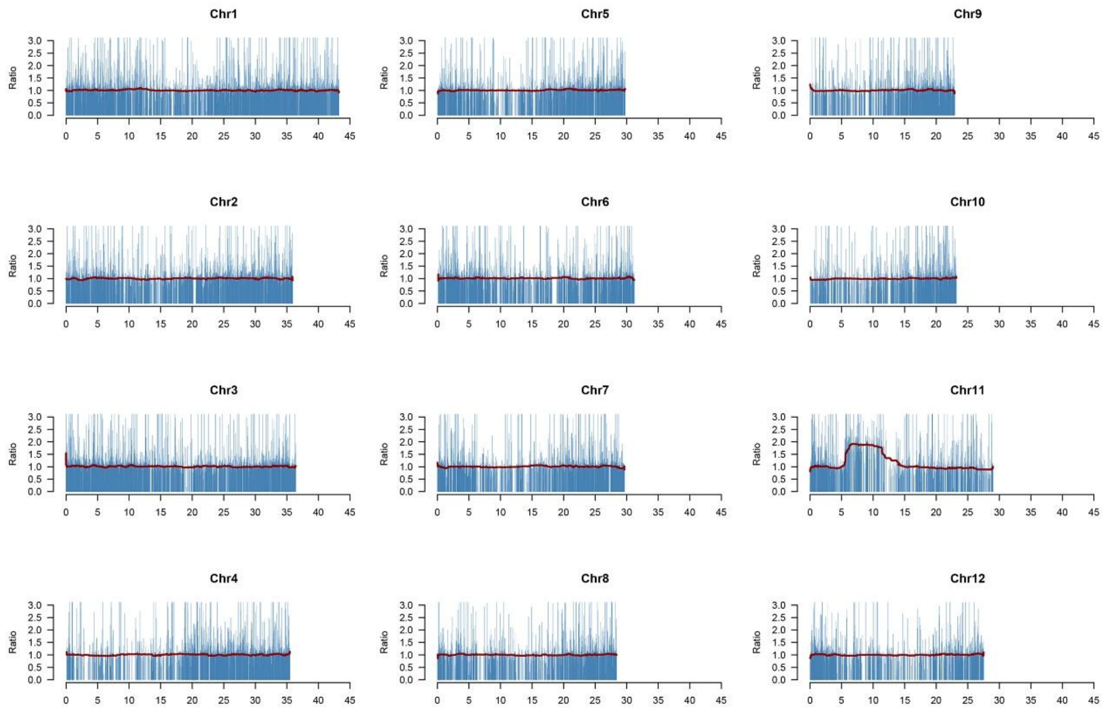


**Figure S3. Gene expression level of each chromosome in YZG-5**

Blue bar represents expression level change of each expressed gene (FPKM>0) in whole rice genome of YZG-5. Red lines represent median score of expression level change in 100 genes sliding windows. X-axis represents genes loci.

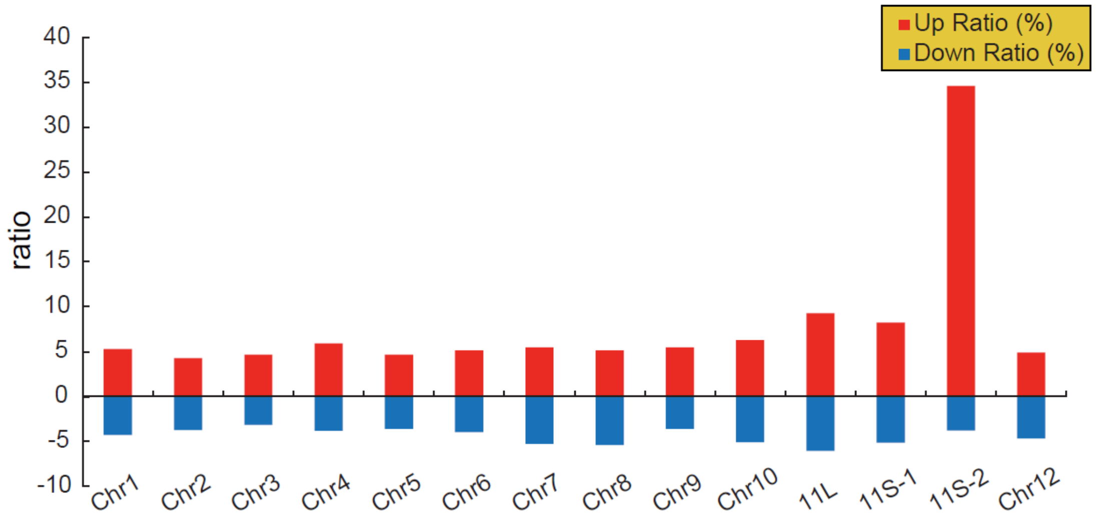


**Figure S4. Ratio of differentially expressed genes to total genes on the corresponding chromosome**

Red bar indicates up-regulated genes, blue bar indicates down-regulated genes. (Ratio=differentially expressed genes/all genes*100)

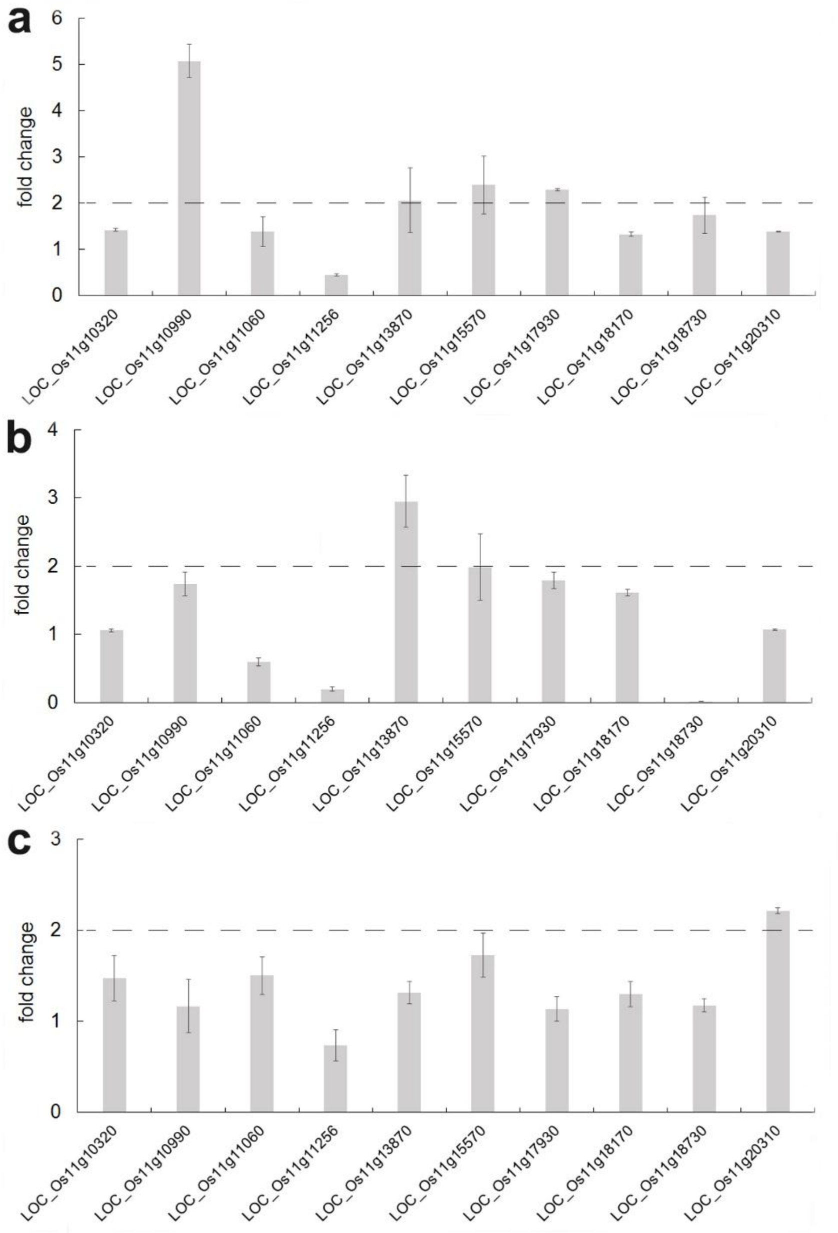


**Figure S5. The expression (fold change) values of ten genes on 11S-2 region in the seedlings of YZG-5 and the seedlings and roots of YZG-5 offsprings**

(**a**) In the seedlings of YZG-5, four genes fold change of expression level are greater than 2.0 and only one gene fold change of expression level is less than 1.0. (**b**) In the leaves of YZG-5 offsprings, except one gene fold change of expression level is greater than 2.0, the remaining are less than 2.0. (**c**) In the roots of YZG-5 offsprings, nine out of ten genes fold change of expression level are less than 2.0.
